# Identification of eight novel *SDHB*, *SDHC*, *SDHD* germline variants in Danish pheochromocytoma/paraganglioma patients

**DOI:** 10.1186/s13053-016-0053-6

**Published:** 2016-06-08

**Authors:** Marc Bennedbæk, Maria Rossing, Åse K. Rasmussen, Anne-Marie Gerdes, Anne-Bine Skytte, Uffe B. Jensen, Finn C. Nielsen, Thomas v. O. Hansen

**Affiliations:** Center for Genomic Medicine, Rigshospitalet, University of Copenhagen, Blegdamsvej 9, DK-2100 Copenhagen, Denmark; Department of Medical Endocrinology, Rigshospitalet, University of Copenhagen, Blegdamsvej 9, DK-2100 Copenhagen, Denmark; Department of Clinical Genetics, Rigshospitalet, University of Copenhagen, Blegdamsvej 9, DK-2100 Copenhagen, Denmark; Department of Clinical Genetics, Aarhus University Hospital, Brendstrupgaardsvej 21 C, Aarhus N, 8200 Denmark

**Keywords:** Classification, Germline, Mutation, Paraganglioma, Pheochromocytoma, *SDHB*, *SDHC*, *SDHD*

## Abstract

**Background:**

Germline mutations in the succinate dehydrogenase complex genes *SDHB*, *SDHC*, and *SDHD* predispose to pheochromocytomas and paragangliomas. Here, we examine the *SDHB*, *SDHC*, and *SDHD* mutation spectrum in the Danish population by screening of 143 Danish pheochromocytoma and paraganglioma patients.

**Methods:**

Mutational screening was performed by Sanger sequencing or next-generation sequencing. The frequencies of variants of unknown clinical significance, e.g. intronic, missense, and synonymous variants, were determined using the Exome Aggregation Consortium database, while the significance of missense mutations was predicted by *in silico* and loss of heterozygosity analysis when possible.

**Results:**

We report 18 germline variants; nine in *SDHB*, six in *SDHC*, and three in *SDHD*. Of these 18 variants, eight are novel. We classify 12 variants as likely pathogenic/pathogenic, one as likely benign, and five as variants of unknown clinical significance.

**Conclusions:**

Identifying and classifying *SDHB*, *SDHC*, and *SDHD* variants present in the Danish population will augment the growing knowledge on variants in these genes and may support future clinical risk assessments.

## Background

Pheochromocytomas (PCC; OMIM 171300) and paragangliomas (PGL; OMIM 115310) are neuroendocrine neoplasms localized in the medulla of the adrenal gland and the extra-adrenal paraganglia, respectively. Both neoplasms stem from the neural crest and are characterized by excess catecholamine production. Approximately 60 % of PCC and PGL are sporadic; however, up to 40 % are due to genetic predisposition [1, 2]. The familial PCC/PGL are associated with germline mutations in at least 11 susceptibility genes, including multiple endocrine neoplasia 2A and 2B (*RET*), von Hippel-Lindau (*VHL*), neurofibromatosis type 1 (*NF1*), transmembrane protein 127 (*TMEM127*), MYC-associated factor X (*MAX*), fumarate hydratase (*FH*), and succinate dehydrogenase complex subunit A, B, C, D, and AF2 (*SDHA*, *SDHB*, *SDHC*, *SDHD*, *SDHAF2*) [3–13]. These genes form the basis for genetic testing and risk assessment in patients suffering from PCC or PGL [1, 2]. Among the first genes to be identified in the tumorigenesis of familial PCC and PGL were *SDHB*, *SDHC*, and *SDHD*, all of which belong to the succinate dehydrogenase gene family [14]. These genes encode a protein complex that is involved in the oxidation of succinate in the respiratory chain and are classified as tumor suppressors. The succinate dehydrogenase complex (complex II) itself is composed of four nuclear-encoded subunits, SDHA, SDHB, SDHC, and SDHD, and functions in the inner mitochondrial membrane where it oxidizes succinate to fumarate. The *SDHB* gene encodes the iron-sulfur catalytic subunit of complex II. It is located on chromosome 1 and comprises eight exons encoding a 280-amino acid protein. The *SDHC* and *SDHD* genes encode the two membrane integral components that anchor the complex to the mitochondrial membrane. The *SDHC* gene is located on chromosome 1 and consists of six exons encoding a protein of 169 amino acids, while the *SDHD* gene is located on chromosome 11 and comprises four exons encoding a protein consisting of 159 amino acids [15–17]. Here, we report the *SDHB*, *SDHC*, and *SDHD* variants identified in Danish PCC and PGL families.

## Methods

### Patients

In agreement with national guidelines in endocrinology (Danish Endocrine Society: http://www.endocrinology.dk/), patients were referred for genetic screening from the Departments of Endocrinology or the Departments of Clinical Genetics throughout the regions of Denmark. After obtaining verbal and written consent from each patient, blood samples were collected for germline variant screening. In total, 143 individuals were screened between 2006 and 2015 for *SDHB*, *SDHC*, and *SDHD* germline variants. This investigation was approved by the local ethics committee in the capital region of Denmark (H-4-2010-050).

### *SDH* screening

Genomic DNA was isolated from whole blood or formalin-fixed paraffin-embedded (FFPE) tumor tissue using a QIAamp DNA mini kit (Qiagen) or QIAamp DNA FFPE tissue kit according to the instructions provided by the manufacturer. From 2006 to 2014, the coding exons and adjacent intronic sequences of *SDHB*, *SDHC*, and *SDHD* were amplified by PCR followed by Sanger sequencing using an ABI3730 DNA analyzer (Applied Biosystems). Moreover, genomic DNA was examined for large genomic rearrangements by multiplex ligation-dependent probe amplification (MLPA) analysis using a SALSA MLPA P226 *SDH* kit (MRC-Holland). Since 2014, the analysis has been performed using targeted next-generation sequencing and a library designed to capture all exons from the three genes. Library construction was carried out from 50–500 ng of genomic DNA and adaptor ligation of Illumina’s adaptors included in the TruSeq DNA sample preparation kit (Illumina) was performed using the SPRIworks System I (Beckman Coulter). Sequence capture was conducted using a double capture protocol (Roche) whereby 8–10 samples were pooled prior to hybridization. Sequencing was performed on a MiSeq (llumina) to an average depth of at least 100×. Sequencing data were analyzed using SequencePilot (JSI medical systems), where variants were called if the allele frequency was above 25 %. Moreover, the samples were analyzed for copy number variations. *SDHB*, *SDHC*, and *SDHD* variants are numbered according to GenBank accession numbers NM_003000.2, NM_003001.3, and NM_003002.3, in which the A in the AUG start codon has number 1, using the guidelines from the Human Genome Variation Society (http://varnomen.hgvs.org/). Sequence variants, except well-known polymorphisms, were verified by Sanger sequencing in a new blood sample.

### *In silico* data analysis

The integrated Alamut Visual software (v.2.6.1) (http://www.interactive-biosoftware.com) including Align GVGD (A-GVGD), PolyPhen-2, and SIFT was used to predict the pathogenicity of specific variants in *SDHB*, *SDHC*, and *SDHD*. The *in silico* effect of variants on splicing was examined as previously described [18]. Default settings were used in all predictions. The frequency of the variants was obtained from the Exome Aggregation Consortium (ExAC) or the Exome Sequencing Project (ESP) databases. Moreover the frequency of novel missense variants was examined in data from 2000 Danish exomes [19]. A combined assessment on the pathogenicity of each variant was used according to the five-tiered scheme, where Class 5 is pathogenic, Class 4 is likely pathogenic, Class 3 is uncertain due to insufficient evidence, Class 2 is likely benign, and Class 1 is benign [20].

## Results

During the last 9 years, we have performed genetic screening of the entire coding region and the exon-intron boundaries of the *SDHB*, *SDHC*, and *SDHD* genes on genomic DNA from Danish PCC/PGL patients. Up until May 2015, we have screened 143 individuals and so far 18 germline variants have been identified, of which eight are novel (Table 1). Moreover, 14 well-known polymorphisms were identified, including six in *SDHB*, five in *SDHC*, and three in *SDHD* (Table 2).

**Table 1:** *SDHB*, *SDHC*, and *SDHD* variants identified in Danish patients with neuroendocrine cancer. *PCC* pheochromocytoma, *PGL* paraganglioma, *LGR* large genomic rearrangement; ^a^ identified in homozygote state; *N/A* not accessible. Results from *In silico* data analyses from A-GVGD, SIFT, and PolyPhen-2 are listed in the corresponding columns. Each variant is classed according to the five-tiered scheme

Family ID	Disease	Age (yr)	Gene	Exon	Nucleotide	Protein	Mutation type	A-GVGD	SIFT	PolyPhen-2	Frequency (ExAC)	Class	References
1	PGL	51	*SDHB*	1	c.-1-?_72+?del	-	LGR	-	-	-	-	5	[21, 22]
2	PGL	36	*SDHB*	1–7	c.-1-?_765+?del	-	LGR	-	-	-	-	5	Novel
3	PGL	29	*SDHB*	1	c.3G > A	p.Met1?	Start loss	-	-	-	-	4	[24, 32, 43]
4	PGL	34	*SDHB*	3	c.203G > A	p.Cys68Tyr	Missense	C65	Deleterious	Possibly damaging	-	4	[30, 43]
5	PGL	16	*SDHB*	4–5	c.287-?_540+?del	-	LGR	-	-	-	-	5	Novel
6	PCC	51	*SDHB*	4	c.300T > C	p.Ser100Ser	Synonymous	-	-	-	0.15 %	3	[30]
7	PCC	29	*SDHB*	4	c.416T > C	p.Leu139Pro	Missense	C65	Deleterious	Possibly damaging	-	4	Novel
8	PGL	12	*SDHB*	6	c.566G > A	p.Cys189Tyr	Missense	C65	Deleterious	Possibly damaging	-	3	Novel
9	PGL	39	*SDHB*	7	c.653G > A	p.Trp218Ter	Nonsense	-	-	-	-	5	[30]
10	PGL	41	*SDHC*	2	c.43C > T	p.Arg15Ter	Nonsense	-	-	-	0.00082 %	5	[31–33]
11	PGL	75	*SDHC*	-	c.78-19C > T	-	-	-	-	-	0.021 %	3	Novel
12	PGL	N/A	*SDHC*	3	c.148C > T	p.Arg50Cys	Missense	C25	Deleterious	Probably damaging	-	4	[32, 43]
13	PGL	64	*SDHC*	4	c.191_207del17	p.Pro64LeufsTer28	Frameshift	-	-	-	-	5	Novel
14	PCC	31	*SDHC*	5	c.242G > T^a^	p.Gly81Val	Missense	C0	Deleterious	Probably damaging	0.00082 %	3	Novel
15	PCC	74	*SDHC*	6	c.496C > G	p.Leu166Val	Missense	C0	Tolerated	Benign	-	3	Novel
16	PGL	41	*SDHD*	2	c.149A > G	p.His50Arg	Missense	C0	Tolerated	Probably damaging	0.65 %	2	[22, 30, 43–48]
17	PCC	37	*SDHD*	3	c.274G > T	p.Asp92Tyr	Missense	C65	Deleterious	Probably damaging	-	5	[4, 36, 38–43]
18	PGL	41	*SDHD*	4	c.341A > G	p.Tyr114Cys	Missense	C65	Deleterious	Probably damaging	-	5	[24, 37, 42, 49–53]

**Table 2:** *SDHB*, *SDHC*, and *SDHD* polymorphisms identified in Danish patients with neuroendocrine cancer

Gene	Nucleotide	Protein	Frequency (ESP)^a^	Frequency (ExAC)^b^
*SDHB*	c.18C > A	p.Ala6Ala	2.70 %	2.99 %
*SDHB*	c.24C > T^c^	p.Ser8Ser	0.76 %	0.65 %
*SDHB*	c.200 + 33G > A	-	7.79 %	8.42 %
*SDHB*	c.200 + 35G > A	-	3.74 %	3.93 %
*SDHB*	c.201-36G > T	-	97.38 %	96.99 %
*SDHB*	c.487T > C	p.Ser163Pro	1.30 %	1.52 %
*SDHC*	c.20 + 11_20 + 12dup	-	6.25 %	6.37 %
*SDHC*	c.77 + 36T > A	-	2.08 %	2.44 %
*SDHC*	c.77 + 43del	-	-	3.15 %
*SDHC*	c.77 + 48C > T	-	5.31 %	4.69 %
*SDHC*	c.406-14del	-	-	41.08 %
*SDHD*	c.170-29A > G	-	1.28 %	1.16 %
*SDHD*	c.204C > T	p.Ser68Ser	1.28 %	1.16 %
*SDHD*	c.315-32T > C	-	1.25 %	1.17 %

The frequency of the polymorphism was obtained from the Exome Sequencing Project (ESP) among ^a^European American and the Exome Aggregation Consortium (ExAC) databases among ^b^European (Non-Finnish); ^c^Observed in 1.35 % of the Finnish population obtained from the ExAC database

**Fig. 1:**
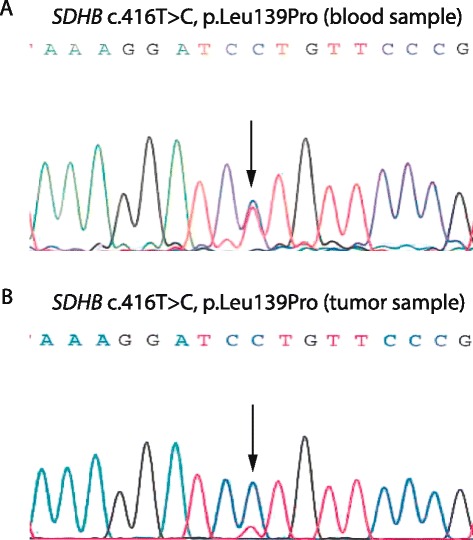
LOH analysis of DNA from the p.Leu139Pro variant carrier. DNA was purified from blood and tumor samples from the patient and *SDHB* exon 4 was amplified using intronic primer pairs flanking exon 4 and sequenced. DNA sequences from blood (**a**) and from tumor tissue (**b**) are shown. The *SDHB* c.416T > C, p.Leu139Pro position is indicated by an *arrow*

Variants in the *SDHB* gene were the most frequent, with a total of nine germline variants of which four are novel. The variants included a p.Trp218Ter nonsense mutation, as well as three large genomic rearrangements (LGR) deleting exon 1 (c.-1-?_72+?del), exons 1–7 (c.-1-?_765+?del), and exons 4–5 (c.287-?_540+?del), of which the two latter are novel. Moreover, we identified a start-loss mutation (p.Met1?) and three missense variants (p.Cys68Tyr, p.Leu139Pro, and p.Cys189Tyr) in SDHB, which were all highly conserved and predicted to be pathogenic by A-GVGD, SIFT, or PolyPhen-2 (Table 1). None of the missense variants have been described before in the ExAC database or in the Danish population. Sequencing analysis of DNA from tumor tissue from the p.Leu139Pro carrier revealed loss of heterozygosity (LOH) (Fig. 1). Finally, we identified a synonymous variant in *SDHB* located in exon 4 (p.Ser100Ser). This variant is reported with a frequency of 0.15 % in the ExAC database.

Moreover, we identified six variants in the *SDHC* gene, including one nonsense mutation, one frameshift mutation, three missense variants, and one intronic variant. Four of the six variants are novel (Table 1). Both the nonsense mutation (p.Arg15Ter) and the frameshift mutation (c.191_207del17) result in premature stop codons. The p.Arg15Ter mutation is reported in one individual out of more than 60,000 (0.00082 %) in the ExAC database. Of the three *SDHC* missense variants (p.Arg50Cys, p.Gly81Val, and p.Leu166Val), only the p.Gly81Val variant is reported in one South Asian individual (0.00082 %) in the ExAC database, while none of the missense variants have been identified in the Danish population. *In silico* analysis predicted that the p.Arg50Cys and p.Gly81Val variants to be pathogenic, while p.Leu166Val was suggested to be benign. Moreover, we identified an intronic variant (c.78-19C > T) in *SDHC*, which is reported with a frequency of 0.021 % in the ExAC database.

Finally, we identified three *SDHD* missense variants (p.His50Arg, p.Asp92Tyr, and p.Tyr114Cys) all of which have previously been described. Two of the missense variants were identified in a single patient (p.His50Arg and p.Tyr114Cys). Only the p.His50Arg variant is reported with a frequency of 0.65 % in the ExAC database. *In silico* analysis predicted the p.Asp92Tyr and p.Tyr114Cys variants to be pathogenic, whereas the p.His50Arg variant was predicted to be benign.

## Discussion

Screening for *SDHB*, *SDHC*, and *SDHD* germline variants in Danish PCC and PGL patients revealed the identification of 18 different germline variants of which eight are novel.

Four of the nine variants identified in *SDHB* are novel, including two LGR. The deletion of exons 1–7 (c.-1-?_765+?del) was identified in a patient who suffered from a PGL at the age of 36, while the deletion of exons 4–5 (c.287-?_540+?del) was identified in a 16-year-old patient diagnosed with PGL. In addition, we found an exon 1 deletion in a patient with PGL and severe hypertension recognized at the age of 51. This deletion has previously been reported in patients with PGL [21, 22]. All LGRs in *SDHB* introduce frameshifts and premature stop codons and were classified as pathogenic (Class 5). The SDHB p.Trp218Ter nonsense mutation was identified in a patient with PGL at the age of 39 and was classified as pathogenic (Class 5) in accordance with a previous report [23]. Moreover, we identified a start-loss mutation (p.Met1?) in SDHB in a patient diagnosed with a PGL at the age of 29. This mutation destroys the methionine start codon and is thought to prevent *SDHB* from being translated from this position. Although an alternative downstream in-frame methionine start codon is present at codon 58, the mutation has previously been associated with the development of hereditary PGL [24]. Furthermore, immunohistochemical (IHC) staining shows loss of SDHB in association with this variant [25] and thus we classify this variant as likely pathogenic (Class 4). All three *SDHB* missense variants are predicted as pathogenic by *in silico* analysis (Table 1). The p.Cys68Tyr variant has previously been described in patients with PCC or PGL, where IHC analysis of tumor tissue showed no SDHB staining or LOH of the wild-type SDHB allele [26, 27]. Based on these findings, the variant is classified as likely pathogenic (Class 4). The p.Leu139Pro variant has not previously been reported. Sequencing analysis of DNA from FFPE tumor tissue showed LOH, indicating that the mutation is likely pathogenic (Class 4). The final missense variant (p.Cys189Tyr) has not previously been reported, although two different amino acid changes at this position (p.Cys189Arg and p.Cys189Phe) have been associated with hereditary PGL [23, 28]. We currently classify this variant as a variant of uncertain significance (VUS) (Class 3) since no co-segregation or functional studies have been performed. Finally, the synonymous variant p.Ser100Ser has previously been reported in patients with PGL [29, 30] but since no functional studies have been performed, we classify this variant as a VUS (Class 3).

The p.Arg15Ter nonsense mutation and the c.191_207del17 frameshift mutation in *SDHC* both result in premature stop codons and are thus classified as pathogenic (Class 5). The p.Arg15Ter variant has previously been reported [31–33], whereas the frameshift mutation is novel. Moreover, we identified three missense variants in *SDHC*, of which p.Gly81Val and p.Leu166Val are novel. *In silico* analysis indicates that the former variant is pathogenic while the latter is predicted to be benign. The nucleotide substitution leading to p.Gly81Val is present on the first base in exon 5 and *in silico* splicing analysis indicates that this change could affect the splicing of exon 5. Recently, another change at position 81 (p.Gly81Arg) has been reported to affect splicing via the splice donor site [23]. The p.Gly81Arg variant was identified in the homozygous state in a patient who developed PCC at the age of 31. Homozygous pathogenic germline mutations usually result in a much more severe phenotype or syndrome, and since no functional studies have been performed at either the RNA or protein level, the p.Gly81Val variant is classified as a VUS (Class 3). The p.Leu166Val variant is located close to the stop codon in *SDHC.* Another variant at position 166 (p.Leu166Arg) has been reported in the LOVD database but is not described in the literature. Since no functional studies have been performed, we classify this variant as a VUS (Class 3). The p.Arg50Cys variant has previously been described, and *in silico* and functional analyses indicate that this variant has some effect on the SDH activity [32, 34]. Moreover, since the variant was identified in an offspring of the proband, who suffered from PGL at a young age (<25 years), the variant is classified as likely pathogenic (Class 4). Finally, we identified a novel intronic variant (c.78-19C > T) in *SDHC* in an elderly patient with PGL at age 75*. In silico* analysis does not predict any effect of this variant on splicing; however, since no functional studies have been performed, it is classified as a VUS (Class 3).

The missense variants identified in *SDHD* have all previously been described. The p.His50Arg variant is predicted to be benign by *in silico* analysis. The variant has been described several times in the literature and functional studies in yeast show that this variant has no effect on the phenotype [35]. In agreement with these data and taking the high frequency in the control population into consideration, we classify the variant as likely benign (Class 2). The p.His50Arg variant was identified alongside a pathogenic mutation p.Tyr114Cys, which is highly conserved and has been shown to co-segregate with disease [4, 34, 36–38] and therefore are classified as pathogenic (Class 5). Finally, the p.Asp92Tyr is a well-known Dutch founder mutation and was indeed identified in a Dutch individual, who had inherited the mutation from his father. Functional analysis has shown that the mutation impairs the function of the SDHD protein [4, 36, 38–42] and in agreement with these findings we classify this variant as pathogenic (Class 5).

In conclusion, we have examined the mutation spectrum associated with familial PCC and PGL in the Danish population. Our investigation uncovered 18 germline variants in *SDHB*, *SDHC*, and *SDHD*, of which eight are novel. Based on the literature, *in silico* data, and LOH analysis, we classify 12 variants as likely pathogenic/pathogenic, one variant as likely benign, while five variants are classified as variants of unknown clinical significance. Further co-segregation or functional studies are needed before these variants can be classified.

## Abbreviations

IHC, immunohistochemical; LOH, loss of heterozygosity; PCC, pheochromocytomas; PGL, paragangliomas; VUS, variant of uncertain significance
